# Update on the pathological roles of prostaglandin E_2_ in neurodegeneration in amyotrophic lateral sclerosis

**DOI:** 10.1186/s40035-023-00366-w

**Published:** 2023-06-19

**Authors:** Hiroshi Nango, Komugi Tsuruta, Hiroko Miyagishi, Yuri Aono, Tadashi Saigusa, Yasuhiro Kosuge

**Affiliations:** 1 Laboratory of Pharmacology, School of Pharmacy, Nihon University, 7-7-1 Narashinodai, Funabashi-Shi, Chiba, 274-8555 Japan; 2 Department of Pharmacology, School of Dentistry at Matsudo, Nihon University, 2-870-1 Sakaechonishi, Matsudo-Shi, Chiba, 271-8587 Japan

**Keywords:** Prostaglandin E_2_, Amyotrophic lateral sclerosis, Motor neuron death, E-prostanoid receptor, Microsomal prostaglandin E synthetase-1

## Abstract

Amyotrophic lateral sclerosis (ALS) is a progressive neurodegenerative disease characterized by selective degeneration of upper and lower motor neurons. The pathogenesis of ALS remains largely unknown; however, inflammation of the spinal cord is a focus of ALS research and an important pathogenic process in ALS. Prostaglandin E_2_ (PGE_2_) is a major lipid mediator generated by the arachidonic-acid cascade and is abundant at inflammatory sites. PGE_2_ levels are increased in the postmortem spinal cords of ALS patients and in ALS model mice. Beneficial therapeutic effects have been obtained in ALS model mice using cyclooxygenase-2 inhibitors to inhibit the biosynthesis of PGE_2_, but the usefulness of this inhibitor has not yet been proven in clinical trials. In this review, we present current evidence on the involvement of PGE_2_ in the progression of ALS and discuss the potential of microsomal prostaglandin E synthase (mPGES) and the prostaglandin receptor E-prostanoid (EP) 2 as therapeutic targets for ALS. Signaling pathways involving prostaglandin receptors mediate toxic effects in the central nervous system. In some situations, however, the receptors mediate neuroprotective effects. Our recent studies demonstrated that levels of mPGES-1, which catalyzes the final step of PGE_2_ biosynthesis, are increased at the early-symptomatic stage in the spinal cords of transgenic ALS model mice carrying the G93A variant of superoxide dismutase-1. In addition, in an experimental motor-neuron model used in studies of ALS, PGE_2_ induces the production of reactive oxygen species and subsequent caspase-3-dependent cytotoxicity through activation of the EP2 receptor. Moreover, this PGE_2_-induced EP2 up-regulation in motor neurons plays a role in the death of motor neurons in ALS model mice. Further understanding of the pathophysiological role of PGE_2_ in neurodegeneration may provide new insights to guide the development of novel therapies for ALS.

## Background

Amyotrophic lateral sclerosis (ALS), also known as Lou Gehrig’s disease, is a devastating neurodegenerative disease characterized by the selective degeneration of upper and lower motor neurons. It causes progressive paralysis and muscle atrophy for which no effective treatment exists, leading to eventual death, usually within 1 to 5 years of onset [1]. Most cases of ALS are sporadic, and the cause of sporadic ALS remains largely unknown. Familial ALS (fALS) accounts for approximately 10% of all cases, and its causative gene defects have been identified, including mutations in genes encoding superoxide dismutase 1 (SOD1), fused in sarcoma, TAR DNA-binding protein-43 (TDP-43), optineurin, and C9ORF72 [2]. Among these etiologies, the best characterized fALS cases are caused by a mutation in the gene encoding SOD1, which accounts for about 20% of fALS cases [3, 4]. The human *SOD1* gene with the G93A point mutation (alanine substitution for glycine at position 93) was the first experimental molecular defect to result in a progressive paralytic disease in transgenic mice with clinical features similar to human ALS [5]. Thus, G93A mice are widely used as a laboratory model to study the pathogenesis and treatment of ALS. Although multiple processes have been implicated in the pathogenesis of ALS, such as RNA processing errors, misfolded proteins, oxidative stress, mitochondrial dysfunction, and glutamate excitotoxicity [4], the mechanism of motor neuron degeneration in ALS remains largely unknown.

The role of spinal-cord inflammation in the progression of ALS is well recognized, and is reflected by the presence of elevated levels of proteins associated with inflammatory states such as phospholipase A_2_ (PLA_2_), cyclooxygenase (COX)-1 and COX-2 [6], and pro-inflammatory cytokines such as interleukin (IL)-6 [7], IL-8 [8], IL-18 [9], and tumor necrosis factor (TNF)-α, in the spinal cord [10]. In particular, growing evidence indicates that the numbers of activated microglia [11] and reactive astrocytes [12] increase in the spinal cords of ALS patients and model mice, indicating that inflammation plays an important role in a vicious cycle of motor neuron degeneration, implicating not only the motor neurons but also the neighboring non-motor neuron cells, especially microglia and astrocytes [13, 14]. Indeed, exogenous SOD1^G93A^ has no direct neurotoxicity to primary motor neurons of mice, and is toxic only when the motor neurons are co-cultured with primary mouse microglia [15]. SOD1^G93A^-activated microglia produce TNF-α, IL-1β, and superoxide [15]. IL-1α, TNF-α, and C1q released from activated microglia contribute to astrocyte activation [16], and a triple deletion of genes for these factors improves motor function and longevity in G93A mice [17]. In addition, increased astrocyte-specific expression of interferon (IFN)-stimulated gene 15 is observed in the spinal cords of G93A mice and ALS patients, and knockdown or knockout of IFN-α receptor 1 prolongs the lifespan of G93A mice [18]. These findings support the hypothesis that inflammation is associated with activation of microglia and astrocytes, and this plays an important role in the progression of ALS. By contrast, single knockout of IL-1β [19], IL-6 [20], or TNF receptor 2 [21] does not affect disease progression in G93A mice, suggesting compensatory mechanisms among these cytokines in ALS progression. An alternative interpretation suggests that the cooperative role of IL-1β, IL-6, and TNF receptor 2 should be important in the progression of ALS, similar to the significance observed with the triple genetic deletion of IL-1α, TNF-α, and C1q [17]. Furthermore, it should be noted that disease progression correlates with increases of different cytokines in cases with different ALS genetic variants [22]. Thus, the inflammatory response in ALS is very complex, and a deeper understanding is required to elucidate the pathogenesis of ALS.

**Fig. 1:**
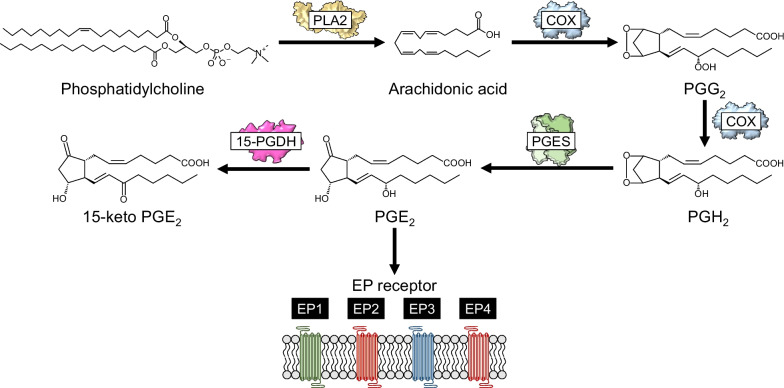
The biosynthetic and metabolic pathways of PGE_2_. COX, cyclooxygenase; EP1-4, E-prostanoid receptor 1–4; 15-PGDH, 15-hydroxyprostaglandin dehydrogenase; PGE_2_, prostaglandin E_2_; PGES, prostaglandin E synthase; PGG_2_, prostaglandin G_2_; PGH_2_, prostaglandin H_2_; PLA_2_, phospholipase A_2_

Prostaglandin E_2_ (PGE_2_) is an eicosanoid lipid metabolite generated by the arachidonic acid cascade via multiple enzymatic reactions [23]. It is increased in postmortem brain tissues, cerebrospinal fluids (CSF), and sera from patients with sporadic ALS [24, 25], and in both the cerebral cortex and spinal cord of G93A mice [26, 27]. PGE_2_ synthesis occurs when arachidonic acid is released from the cell membrane specifically by cytosolic PLA_2_ (cPLA_2_) and is metabolized by COX-1 and COX-2 to produce prostaglandin H_2_ (PGH_2_) [28]. Subsequently, PGH_2_ is converted to PGE_2_ by prostaglandin E synthase (PGES) [29, 30]. At least three distinct types of PGESs have been identified and characterized, namely, cytosolic PGES (cPGES) [31], microsomal PGES (mPGES)-1 [32], and mPGES-2 [33]. cPGES and mPGES-2 are constitutively expressed and involved in the physiological production of PGE_2_, while mPGES-1 is inducible under inflammatory conditions and is functionally coupled to COX-2 [34]. The newly synthesized PGE_2_ binds to specific G-protein-coupled receptors (i.e., E-prostanoid receptors [EP] 1 to 4) at the cell-surface membrane, where it exerts its physiological functions [29, 30]. Functionally, EP1 increases intracellular Ca^2+^ levels via G_αq_-dependent activation of phospholipase C; EP2 and EP4 increase intracellular levels of cyclic adenosine monophosphate (cAMP) via G_αs_-dependent activation of adenylyl cyclase; and EP3 increases or decreases intracellular cAMP via G_αs_ or G_αi_, respectively [35]. The binding affinities of PGE_2_ to the EPs are as follows: EP3 (*K*_i_ = 0.9 nM) > EP4 (*K*_i_ = 1.9 nM) > EP2 (*K*_i_ = 12 nM) > EP1 (*K*_i_ = 20 nM) [36]. Free PGE_2_ is rapidly oxidized to 15-ketoPGE_2_ by 15-hydroxyprostaglandin dehydrogenase (15-PGDH), followed by reduction of the Δ^13^ double bond by Δ^13^-15-keto prostaglandin reductase and β-oxidation, resulting in biological inactivation [37, 38]. 15-PGDH is the key enzyme in the biological inactivation of PGE_2_, catalyzing the first step. To date, there are two known isoforms of 15-PGDH: NAD^+^-dependent (type-I) and NADP^+^-dependent (type-II) [39]. Type I has a *K*m value of 8.60, whereas type II has a *K*m value of 81.1 [40], suggesting that the type I 15-PGDH plays a major role in the catabolism of PGE_2_. Figure 1 shows the major biosynthetic and metabolic pathways of PGE_2_. To date, accumulatig evidence demonstrates that PGE_2_ can have opposite effects on neurons, either contributing to neuroprotection or enhancing neuroinflammatory and neurodegenerative processes [41]. Understanding the pathological function of PGE_2_ in ALS will allow development of new therapies.

In this paper, we summarize the roles of PGE_2_-related enzymes and receptors in ALS and the effects of PGE_2_ on motor-neuron death, and discuss the potential of PGE_2_ as a target for therapeutic regeneration in ALS.

### Roles of the mPGES-1-dependent synthetic pathway and the 15-PGDH-dependent catabolic system in ALS

The expressions of cPLA_2_ and COX-2 are up-regulated in the spinal cords of ALS patients and ALS model mice [6, 42–44]. Nimesulide, a COX-2-selective inhibitor, suppresses PGE_2_ elevation in the spinal cord, delays the onset of motor deficits, and tends to prolong the survival of G93A mice [45]. Furthermore, inhibition of COX-2 in G93A mice by celecoxib or rofecoxib, which are more selective than nimesulide, reduces the production of PGE_2_, delays the onset of weight loss, ameliorates motor performance degeneration, extends survival, and protects against depletion of motor neurons in the spinal cord [26, 46]. We previously discovered that the protein level of mPGES-1 in the spinal cord was significantly increased after 15 weeks of age, which represents the early symptomatic stage, while the levels of cPGES and mPGES-2 remained constant with age [47]. Similar to the increased expression of mPGES-1, we found that the protein level of Iba1, a microglial marker, was also significantly increased in the G93A mice after 15 weeks of age. Surprisingly, our immunofluorescence staining assay demonstrated that mPGES-1 was co-localized with motor neurons both at 11 weeks (a pre-symptomatic stage) and at 15 weeks of age in these mice. On the contrary, co-localization of mPGES-1 with Iba1-positive microglia was detected in the spinal cords of G93A mice only at 15 weeks. Unlike the microglial marker Iba1, expression of the astrocytic marker glial fibrillary acidic protein (GFAP) was increased at 17 and 19 weeks of age (the symptomatic and end stage, respectively), with little co-localization with mPGES-1. These results suggest that, at the early symptomatic stage, mPGES-1, but not cPGES or mPGES-2, contributes to the PGE_2_ increase in activated microglia in the spinal cord and to the progression of motor neuron degeneration in ALS.

Interestingly, AAD-2004, a dual-function drug derived from 5-aminosalicylate and sulfasalazine, has been reported to prevent PGE_2_ production, microglial activation, and motor neuron degeneration through blockade of both mPGES-1 and free-radical production in G93A mice, resulting in amelioration of motor function and prolonged survival [48]. Moreover, the therapeutic effect of AAD-2004 is superior to that of ibuprofen, a type of non-steroidal anti-inflammatory drug (NSAID) [48]. While these results seem to support ours, it should be noted that the therapeutic effect of AAD-2004 is not only dependent on the inhibition of mPGES-1, but also requires direct free-radical scavenging. Deletion of mPGES-1  has been reported to suppress oxidative stress in angiotensin II-treated hyperlipidemic mice [49] and in 6-hydroxydopamine (6-OHDA)-treated mice [50]. These reports make it difficult to determine the degree to which free-radical scavenging contributes to the therapeutic efficacy of AAD-2004, and thus it is worth investigating the therapeutic effects of mPGES-1 inhibitors without direct free-radical scavenging activity in ALS, in comparison with the effects of combination of NSAIDs and antioxidants. In addition, AAD-2004 lowers  PGE_2_ levels without affecting the production of prostacyclin (PGI_2_) in the lumbar spinal cord of G93A mice [48]. Considering that a sustained-release PGI_2_ receptor agonist, ONO-1301-MS, improves motor function and prevents loss of motor neurons in G93A mice [51], the selective inhibition of PGE_2_ production by mPGES-1 is more beneficial for the treatment of ALS than the non-selective inhibition of PG production by COX-2.

Our study described above revealed the involvement of synthetic steps of PGE_2_ in ALS [47]. With regard to the degradation process of PGE_2_, our previous study [27] detected a band immunoreactive for 15-PGDH in the spinal cord of G93A mice starting at 15 weeks (the early symptomatic stage), and its expression was significantly increased at 17 weeks and older. Immunohistochemical analysis showed that 15-PGDH was co-localized with GFAP-positive astrocytes at 19 weeks. Unlike mPGES-1, 15-PGDH did not co-localize with NeuN-positive motor neurons or Iba-1-positive microglia in the spinal cord of G93A mice. Surprisingly, despite an increase in 15-PGDH in astrocytes, PGE_2_ levels in the lumbar spinal cord were increased in an age-dependent manner, with a significant increase at 19 weeks. These results suggest that the production of PGE_2_ overwhelms the increase of 15-PGDH and its activity in astrocytes at the end stage of ALS. An in vitro study reported that the uptake of PGE_2_ via the prostaglandin transporter, an organic anion transporter, as well as 15-PGDH, is required for PGE_2_ catabolism [52]. Although further studies are required to determine the expression of the prostaglandin transporter in spinal astrocytes in G93A mice, these results suggest that astrocytes expressing 15-PGDH internalize PGE_2_ synthesized by mPGES-1-expressing motor neurons and microglia to maintain PGE_2_ homeostasis.

**Fig. 2:**
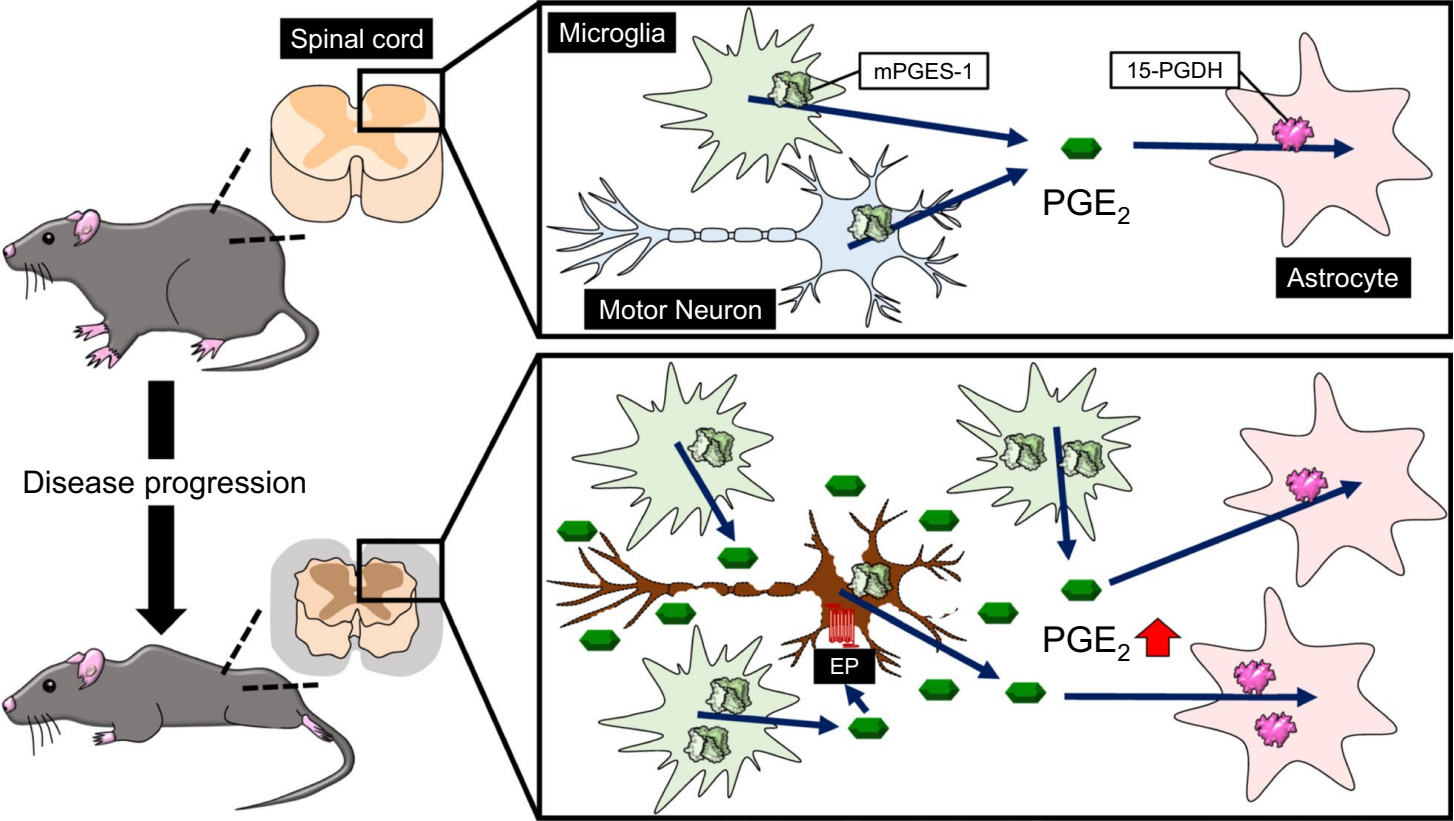
Changes in PGE_2_ synthesis and metabolism during disease progression in G93A model mice. EP, E-prostanoid receptor; mPGES-1, microsomal prostaglandin E synthase-1; 15-PGDH, 15-hydroxyprostaglandin dehydrogenase; PGE_2_, prostaglandin E_2_

Combining these results with those of our studies [27, 47], we offer a new concept that a significant increase of PGE_2_ level is attributable to an imbalance between the mPGES-1-dependent synthetic pathway and the 15-PGDH-dependent scavenging system in ALS model mice (Fig. 2). Unlike our results from G93A mice, a decrease in the expression or activity of 15-PGDH correlates with an increase of PGE_2_ in the hypothalamus of a rat model of chronic unpredictable mild stress [53] and in ischemic regions including cortex, striatum, and hippocampus in a rat stroke model (middle cerebral artery occlusion-reperfusion) [54]. Therefore, an imbalance in the synthesis and catabolism of PGE_2_, despite an increase in 15-PGDH, may be the pathogenic mechanism of the transition from the early-symptomatic stage of ALS or motor-neuron disease. However, currently there have been no reports on the role of mPGES-1 and 15-PGDH in ALS patients, even in patients with other neurodegenerative diseases. Therefore, further investigation of their clinical significance is warranted.

### The pathophysiological role of EPs in motor neurons in ALS

PGE_2_ exerts its physiological functions by binding to the EPs. The activation of EP1 contributes to neurotoxicity in cultured rat mesencephalic neurons [55]. EP2 has been shown to induce caspase-3–dependent cell death in cultured rat cortical and hippocampal neurons [56, 57]. EP3 activation mediates the glutamate-induced excitotoxicity in cultured rat hippocampal slices [58]. In contrast, EP2 protects against glutamate cytotoxicity in cultured rat hippocampal neurons [59]. Furthermore, EP2 and EP3 have a protective effect against glutamate cytotoxicity induced by glutamate transporter inhibition in rat organotypic spinal cord slice cultures [60]. The EP4 receptor agonist ONO-AE1-329 attenuates injury in the brain in an acute murine model of the *N*-methyl-*D*-aspartate-induced excitotoxicity [61]. These studies suggest that PGE_2_ exerts protective or pathogenic effects against neurotoxicity, depending on the subtype or the cell target of the activated EP. The NSC-34 cell line is the most studied motor-neuron cell line, and is produced by the fusion of mouse neuroblastoma cells with motor neurons taken from embryonic spinal cords [62]. Differentiated NSC-34 cells are reported to exhibit the unique morphological and physiological characteristics of primary motor neurons [62, 63]. Our group was the first to identify the distribution of EP subtypes and their effects on the PGE_2_-induced cell death in differentiated NSC-34 cells [64]. EP2 and EP3 were highly expressed in mouse spinal cord motor neurons and in differentiated NSC-34 cells. Exposure of NSC-34 cells to exogenous PGE_2_ and butaprost, an EP2-selective agonist, resulted in decreased cell viability in a concentration-dependent manner, whereas exposure to sulprostone, an EP1 and EP3 agonist, did not. These results suggest that activation of EP2, but not EP3, contributes to the PGE_2_-induced motor-neuron death.

In support of our results [64], genetic deletion of EP2 has been reported to down-regulate the pro-inflammatory process, improve motor strength and extend survival of G93A mice [65], suggesting that the PGE_2_–EP2 axis plays an important role in ALS neurodegeneration. Liang et al. also revealed that the expression of EP2 was increased in GFAP-positive astrocytes and Iba1-positive microglia of spinal cord [65]. We demonstrated that the EP2 immunoreactivity that co-localized with motor neurons in G93A mice at 15 weeks (the early symptomatic stage) was significantly more intense than that in age-matched wild-type mice and than that at 11 weeks (the pre-symptomatic stage) [66]. An in vitro study using differentiated NSC-34 cells showed that PGE_2_ up-regulated the expression of EP2. The cells with an increased level of EP2 resulting from pre-treatment with PGE_2_ were significantly more vulnerable to the PGE_2_-induced cell death. Consistently, PGE_2_ is involved in the up-regulation of EP1 and EP4 in dorsal root ganglion neurons from partial sciatic nerve-ligated rats [67] and in the up-regulation of EP1 and EP2 in primary rat hepatocytes [68]. Although the mechanism underlying the positive-feedback regulation of EP2 in vivo has yet to be clarified, these results suggest that PGE_2_ increases the expression of EP2 in motor neurons during the early symptomatic stage of ALS, and that its increased expression directly drives neurodegeneration in ALS.

**Fig. 3:**
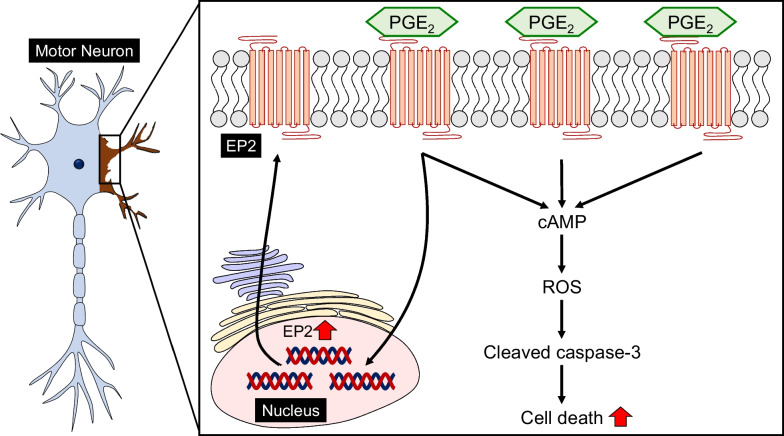
A proposed mechanism underlying the PGE_2_-induced motor neuron degeneration. cAMP, cyclic adenosine monophosphate; EP2, E-prostanoid receptor-2; PGE_2_, prostaglandin E_2_; ROS, reactive oxygen species

A growing body of evidence implicates the dysfunction of cellular reduction/oxidation, termed oxidative stress, in the pathogenesis of ALS [69]. Indeed, edaravone, a free-radical scavenger, was approved as a therapeutic drug for ALS in 2015 in Japan and South Korea, and in 2017 in the United States [70]. The most important point is that oxidative stress causes inflammatory responses, and conversely, uncontrolled inflammation leads to cellular accumulation of reactive oxygen species (ROS), which are involved in oxidative stress [71, 72]. ROS activate a wide range of signaling pathways, such as nuclear factor κB, activator protein-1, and CBP/p300, and thus have been implicated in the induction of expression of cPLA_2_, COX-2, and mPGES-1 [73]. IL-1β induces the up-regulation of PGE_2_ through ROS production in primary human synovial fibroblasts, a process mediated by cPLA_2_ [74]. Induction of COX-2 expression by IL-1, TNF-α, and lipopolysaccharide (LPS) also mediates ROS accumulation in rat mesangial cells [75]. By contrast, genetic deletion of mPGES-1 has been found to prevent the 6-OHDA-induced production of PGE_2_ and oxidative stress-mediated neurotoxicity in dopaminergic neurons of the substantia nigra of mice, and that exogenous application of PGE_2_ to mPGES-1-knockout neurons reverses the 6-OHDA-induced production of PGE_2_ and neurotoxicity [50]. This suggests that PGE_2_ plays an important role in ROS induction in oxidative stress-mediated neuronal death. Consistent with this report, our recent study demonstrated that exposure of differentiated NSC-34 cells to PGE_2_ resulted in a significant increase in intracellular ROS levels [76]. Pre-treatment with *N*-acetyl-*L*-cysteine (NAC), which is widely used as a pharmacological antioxidant, prevented the PGE_2_-induced increases in intracellular ROS production and cleaved caspase-3, resulting in increased cell viability. Furthermore, similar to the results with PGE_2_, butaprost, an EP2-selective agonist, induced an increase in the generation of intracellular ROS and cleaved caspase-3, and these effects were inhibited by NAC. By contrast, sulprostone, an EP1 and EP3 agonist, transiently decreased the level of intracellular ROS. We also showed that PF-04418948, an EP2-selective antagonist, suppressed the PGE_2_-induced generation of intracellular ROS. Moreover, dibutyryl-cAMP, a cell-permeable cAMP analog, partially mimicked the PGE_2_- and butaprost-induced stimulation of intracellular ROS and neurotoxicity in differentiated NSC-34 cells [76]. Based on these results, we conclude that in differentiated NSC-34 cells, PGE_2_ activates EP2 to induce caspase-3-dependent apoptosis by the production of intracellular ROS, and that an increase in intracellular cAMP is at least partly involved as a downstream effect of the PGE_2_–EP2 pathway. In support of our results, deletion of EP2 results in a significant decrease in F4-neuroprostanes, specific markers of oxidative injury to neuronal elements, in APPSwe/PSEN1dE9 mice [77] and G93A mice [65]. Moreover, the cAMP-dependent protein kinase A signaling pathway is reported to be activated under hypoxic conditions and exacerbate hypoxia-induced ROS formation in PC12 cells [78]. However, another study demonstrated that butaprost is protective against 6-OHDA-mediated oxidative stress through cAMP-dependent protein kinase A activity in cultured primary dopaminergic neurons prepared from embryonic rat midbrain [79]. Although further studies are required to identify the downstream mechanisms of the EP2–cAMP axis responsible for ROS production by PGE_2_, our studies demonstrated that PGE_2_ has the ability to induce direct neurotoxicity via the EP2–cAMP–ROS axis in motor neuron-like NSC-34 cells. The mechanism of the PGE_2_-induced cell death in differentiated NSC-34 cells is summarized in Fig. 3.

Mouse EP3 has three distinct splicing variants: the EP3α and the EP3β isoforms are coupled to G_αi_ and the EP3γ isoform is coupled to both G_αs_ and G_αi_ [80]. In our previous study, the expression of EP3α and EP3γ, but not EP3β, was detected in the spinal cords of wild-type and G93A mice [81], and the expression was not significantly different between wild-type and G93A mice. Next, laser-capture microdissection was performed to dissect out motor neurons from samples of lumbar spinal cord. In the motor neurons, EP3γ mRNA expression was predominant in both wild-type and G93A mice, whereas EP3α and EP3β mRNAs were not detected. Similar to the results in spinal cords, the expression level of EP3γ in motor neurons did not differ between G93A and wild-type mice [81]. Furthermore, EP3γ has predominant mRNA expression in differentiated NSC-34 cells [76]. Our previous studies showed that activation of EP3, unlike EP2, failed to contribute to PGE_2_-induced cell death in motor neurons [64, 66, 76]. However, another study using rat organotypic spinal cord slice cultures showed that sulprostone had a protective effect against glutamate neurotoxicity [60]. Therefore, further investigations are warranted to determine the effect of EP3 on neuronal cell death induced by species other than PGE_2_ and associated with the progression of ALS.

### Possible crosstalks between pro-inflammatory cytokines and PGE_2_ in ALS

mPGES-1, 15-PGDH, and EP2 have been shown to be induced in the spinal cords in G93A mice. However, the underlying mechanism of the induction in ALS remains to be determined. One possible contributor to the induction of these expressions is pro-inflammatory cytokines. The spinal cords of mice injected with either IL-1β or TNF-α show increased expression of COX-2 [82]. TNF-α induces PGE_2_ synthesis via increased expression of COX-2 and mPGES-1 in primary mixed spinal cord cells from mice [83]. Polyinosinic-polycytidylic acid increases PGE_2_ production through induction of COX-2 and mPGES-1 synthesis in primary rat microglia [84]. In primary mouse astrocytes, PGE_2_ expression can be induced by TNF-α [85] and IL-1β stimulation [86]. Although the mechanism of 15-PGDH expression in the nervous system is unknown, IL-4 has been reported to increase it in human lung cancer cells [87]. Furthermore, IL-1β increases the expression of EP2 and EP4 in primary rat hippocampal neurons [88], and EP3 expression in primary rat astrocytes and the U373 human astrocyte cell line [89]. However, in another study, IL-1β and TNF-α did not alter the mRNA expression of EP receptors in primary rat dorsal root ganglia cells [90], suggesting that the regulation of EP expression by proinflammatory cytokines depends on the cell type. These studies indicate that pro-inflammatory cytokines induce up-regulation of mPGES-1, 15-PGDH, and EPs under pathological conditions, and further studies are required.

Interestingly, however, PGE_2_ suppresses the LPS-induced COX-2 and mPGES-1 expression and decreases TNF-α production via activation of EP2 in primary mouse mixed spinal cord cells [91]. Studies using the BV-2 mouse microglial cell line have also reported that PGE_2_ suppresses the LPS-induced IL-18 expression [86] and the amyloid-β (Aβ)-induced TNF-α expression [92]. The latter may be mediated by EP4 [92]. These reports support for a protective role of PGE_2_, contrary to our studies. Considering that COX-2-selective inhibitors [26, 45, 46] and a mPGES-1 inhibitor [48] suppress disease progression in G93A mice, the responsiveness to PGE_2_ may differ between LPS-stimulated transient inflammatory conditions and the chronic inflammatory conditions of ALS. Most importantly, previous studies have reported that the primary astrocytes from G93A mice [85] and TDP-43–deficient microglia [93] show upregulation of COX-2 expression and PGE_2_ production. G93A mouse-derived primary astrocytes have higher levels of basal TNF-α and elicit a greater increase in TNF-α expression after either a IFN-γ or a TNF-α challenge compared with those from non-transgenic mice [85]. Therefore, although further investigations are required, the chronic increase in basal levels of PGE_2_ in SOD1^G93A^ mice may facilitate the transition of astrocytes into a more active inflammatory state. Furthermore, TDP-43-deficient microglia induced neuronal death in a co-culture system, which was suppressed by the COX-2 inhibitor celecoxib [93], suggesting that the glial cells with genetic variant associated with ALS, not limited to SOD1^G93A^, contribute to neurodegeneration in ALS through elevated basal levels of PGE_2_.

### Limitations due to the failure of a clinical study and the potential of PGE_2_ as a differentiation factor

We here examine the role of the PGE_2_-related enzymes and receptors in ALS and the cytotoxicity and interactions of PGE_2_ relevant to motor neurons, and we discuss the importance of PGE_2_ for the progression of ALS. This review has some limitations, but it also has some important implications for future studies.

First, even though COX-2 inhibitors improve motor function and prolong the life span of G93A mice [26, 45, 46], no anti-inflammatory drugs, including COX-2 inhibitors, have shown significant benefits in clinical studies in ALS patients [94]. The administration of celecoxib at 800 mg/day for 12 months did not improve muscle strength, vital capacity, estimated exercise units, or the ALS Functional Rating Scale-Revised, nor did it prolong survival [95]. The most notable issue is an absence of PGE_2_ increase in the CSF of ALS patients, and that celecoxib did not reduce PGE_2_ levels in the CSF of ALS patients [95]. However, other studies have reported increased levels of PGE_2_ in the CSF of sporadic ALS patients [24, 25]. For instance, Cudkowicz et al. suggested that the failure to detect high levels of PGE_2_ was due to the absence of a significant degree of inflammation at the disease stage studied, or due to the fact that PGE_2_ levels in the CSF do not necessarily reflect COX-2 activity in the central nervous system [95]. This discrepancy may be explained by the expression and localization of mPGES-1 in the spinal cord [48]. Although the measured concentrations in clinical studies refer to levels in the CSF, the local tissue concentrations are more important because PGE_2_ acts as an autocrine or paracrine factor in target cells. We observed that mPGES-1 co-localizes with motor neurons and activated microglia of the spinal cord [48], suggesting that the local up-regulation of mPGES-1 under pathological conditions contributes to increased local concentrations of PGE_2_ and that PGE_2_ can be produced locally even under COX-2 inhibition. Thus, inhibition of mPGES-1 or antagonism of EP2 remains useful therapeutic targets. However, the local cellular concentrations of PGE_2_ in the spinal cord are unknown. Furthermore, no studies have tested mPGES-1 inhibitors [96] or EP2 antagonists in ALS patients, so their usefulness in humans is unclear; further studies are required. Most recently, it has been reported that the combination of ciprofloxacin and celecoxib restores the morphological defects and abnormal neuromuscular junctions of motor neurons and improves locomotor function in SOD1 G93R transgenic zebrafish and TDP-43-mutant zebrafish, compared to treatment with each drug alone [97]. Therefore, the potential synergistic effects of COX-2 inhibitors with other pharmaceuticals are of interest.

Second, the PGE_2_–EP2–cAMP signaling pathway may be involved not only in motor-neuron cell death but also in motor-neuron differentiation [98]. We previously showed that PGE_2_ induces morphological differentiation through EP2 activation in undifferentiated NSC-34 cells. Moreover, the cAMP signaling axis is at least partially involved in the molecular mechanisms of these effects [99]. Furthermore, it is noteworthy that PGE_2_-differentiated cells have the physiological and electrophysiological properties of mature motor neurons [100]. Previous studies have reported that the COX-2 inhibitors meloxicam and nimesulide suppress neurogenesis in the olfactory bulb in the adult mouse brain [101], and that PGE_2_ promotes the neural differentiation of mouse brain-derived neuroectodermal stem cells [102], suggesting that PGE_2_ plays a key role in the differentiation of endogenous neural stem/progenitor cells into neural cells. Interestingly, neural progenitor cells were reported to migrate to regions near degenerated motor neurons in the spinal cord of G93A mice during the early symptomatic and symptomatic stages and then differentiate into neurons [103]. According to discussions of mPGES-1 in the first limitation, high concentrations of PGE_2_ are present near degenerated motor neurons. Although further studies using adult neural progenitor cells derived from the spinal cord are required to clarify the role of PGE_2_ in the differentiation of motor neurons, these shreds of evidence suggest that PGE_2_ may also play an important role in a neurogenesis process that compensates for the neurodegeneration of ALS. Taken together, these results suggest new physiological roles for PGE_2_, where it can exert multiple effects depending on the stage of neurodevelopment of spinal motor neurons. This supports the hypothesis that increased PGE_2_ in the spinal cord, the mechanistic focus of ALS, is induced to promote the differentiation of new motor neurons, but the net effect is toxicity to surrounding mature motor neurons, resulting in the exacerbation of ALS.

### The pathophysiological roles of PGE_2_ in other neurodegenerative diseases

The level of PGE_2_ in the central nervous system is also increased in other neurodegenerative disorders, such as in the CSF in Alzheimer’s disease (AD) [104] and in the substantia nigra in Parkinson's disease (PD) [105].

AD is the most common cause of dementia/memory loss [106], characterized by accumulation of Aβ and neuronal tau proteins in the brain, which leads to the destruction of cholinergic neurons and damage to brain tissue. In the brains of AD patients, COX-1 accumulates mainly in microglia, whereas COX-2 accumulates mainly in neurons [107]. Furthermore, immunofluorescence staining of AD brain tissues showed that the expression of mPGES-1 was increased in neurons, microglia, astrocytes, and endothelial cells [108], and that mPGES-2 was markedly increased in pyramidal neurons [109]. Clinical trials have reported that treatment with indomethacin, a nonselective COX inhibitor, ameliorates cognitive impairment in patients with AD [110], and that naproxen, a selective COX-2 inhibitor, blocks the Aβ-mediated suppression of long-term potentiation and memory function in Tg2576 AD model mice [111]. Interestingly, PGE_2_ induces the production of Aβ through activation of EP2 and EP4 both in vitro and in vivo [112, 113]. Genetic deletion of EP2 prevents oxidative brain damage, reduces Aβ levels, and improves the spatial memory of AD model mice [77, 114]. Unfortunately, as of May 27, 2023, there have been no clinical trials on AD that target the enzymes or receptors associated with PGE_2_ [115].

Huntington’s disease (HD) is an autosomal dominant pathological disease characterized by a movement disorder and dementia [116]. Although the expression and distribution of PGE_2_-related enzymes and receptors in HD are poorly understood, in vivo studies in R6/1 HD model mice showed that antagonization of EP1 by SC-51089 ameliorated memory and motor deficits [117], while activation of EP2 by misoprostol ameliorated long-term memory deficits through up-regulation of brain-derived neurotrophic factor in the hippocampus [118]. These reports suggest that EP could be a promising therapeutic target; to our knowledge, there has been no report on the clinical significance of PGE_2_ in HD, so further investigation is warranted.

PD is characterized by abnormal α-synuclein aggregations known as Lewy bodies and Lewy neurites, resulting in bradykinesia due to massive death of the nigrostriatal dopaminergic neurons [119]. Consistent with increased PGE_2_ [105], the expressions of COX-2 and mPGES-1 are up-regulated in the substantia nigra of post-mortem PD brains compared to those of healthy controls [50, 120]. Previous studies in the 1-methyl-4-phenyl-1,2,3,6-tetrahydropyridine-induced mouse model of PD showed that DuP697, a selective COX-2 inhibitor, significantly reduced PGE_2_ production and dopaminergic neurotoxicity [121], and that COX-2 deficiency combined with the COX-2-specific inhibitor valdecoxib attenuated the microglial activation, the loss of dopaminergic neurons, and the motor deficits [122]. However, a nested case–control study found no benefit of NSAIDs in PD [123]. Conversely, a meta-analysis by Gao et al. demonstrated that ibuprofen, unlike other NSAIDs or acetaminophen, lowers the risk of early PD progressing to PD compared to non-administration [124]; thus the neuroprotective effect of ibuprofen is noteworthy. In vitro studies reported that activation of EP1 was selectively neurotoxic to dopaminergic neurons in rat embryonic primary mesencephalic neuronal cultures [55], and that activation of EP2 by butaprost protected against the 6-OHDA-induced oxidative stress in midbrain-derived dopaminergic neurons from embryonic rats [79]. By contrast, ablation of EP2 enhanced microglia-mediated α-synuclein clearance in a co-culture of mouse microglia and mesocortical slices from patients with Lewy body disease. Ablation of EP2 also significantly reduced the level of neurotoxicity and α-synuclein aggregation in mice treated with 1-methyl-4-phenyl-1,2,3,6-tetrahydropyridine [125]. Because of these conflicting results [79, 125], the therapeutic effects of EP2 on PD warrant further investigation.

Multiple sclerosis (MS) is an autoimmune disease characterized by white-matter lesions attributable to loss of the myelin sheaths around the axons of neurons [126]. Although PGE_2_ levels in MS patients are unknown, elevated expression of COX-1 and COX-2 and levels of PGE_2_ have been observed in the cerebral cortex, cerebellum, and spinal cord of MS model mice with experimental autoimmune encephalomyelitis (EAE) [127]. In support of this, indomethacin, a non-selective COX inhibitor, attenuates the progression of EAE [128]. Furthermore, mPGES-1 upregulation occurs in macrophages in the spinal cord lesions of the EAE mouse model and in the brain tissues of MS patients [129]. mPGES-1-deficient mice have better clinical scores and suppressed responses of T helper 1 and helper 17 cells compared with non-deficient mice after EAE induction [129]. In EAE mice, PGE_2_ has two opposing effects: it promotes the generation of T helper 1 and helper 17 cells through EP2 and EP4 while protecting the blood–brain-barrier via EP4 to prevent invasion of these cells into the brain [130].

Taken together, these studies indicate that PGE_2_ is strongly correlated with the significant neuroinflammation characterizing the pathogenesis of neurodegenerative diseases, including ALS (Table 1).

**Table 1:** The pathophysiological roles of PGE_2_ and its related enzymes and receptors in ALS and other neurodegenerative diseases

	Enzymes				Receptors			
	PGE_2_	COX	PGES	15-PGDH	EP1	EP2	EP3	EP4
ALS	*Increase* Model mice: cerebral cortex and spinal cord [26, 27] Patients: brain tissue, CSF, and serum [24, 25]	*Upregulation* Model mice: COX-2 in spinal cord [45]; Patients: COX-2 in spinal cord [6] *Treatment* Model mice: Nimesulide—Delay in the onset of movement disorders and tendency to prolong survival [45] Celecoxib —Delay in the onset of movement disorders and prolong survival [26, 46] Rofecoxib —Delay in the onset of movement disorders and prolong survival [26] Patients: Celecoxib —No effect [94]	*Upregulation* Model mice: mPGES-1 in motor neurons and microglia [47]	*Upregulation* Model mice: astrocytes [27]	Unknown	*Upregulation* Model mice: Astrocytes [65], microglia [65] and motor neurons [66] *Treatment* Model mice: Knockout—Prolong survival [65], and suppression of oxidative injury [65]; Model cells: Butaprost (agonist)—Caspase-3-dependent apoptosis via ROS production [76]	*No change* Model mice: Motor neurons [81]	Unknown
AD	*Increase* Patients: CSF [104]	*Upregulation* Patients: COX-1 in microglia, and COX-2 in neurons [73] *Treatment* Model mice: Naproxen—Improvement of memory function[111] Patients: Indomethacin—Improvement of cognitive impairment [110]	*Upregulation* Patients: mPGES-1 in neurons, microglia, astrocytes, and endothelial cells [108]; mPGES-2 in pyramidal neurons [109]	Unknown	Unknown	*Treatment* Model mice: Knockout − Suppression of oxidative injury [77, 114]; Model cells: AE-259 (agonist) − Enhanced Aβ production [113]	Unknown	*Treatment* Model cells: AE-329 (agonist)—Enhanced Ap production [113]
HD	Unknown	Unknown	Unknown	Unknown	*Treatment* Model mice: SC-51089 (antagonist)—Improvement of memory and motor deficits [119]	*Treatment* Model mice: Misoprostol (agonist)—Improvement of longterm memory deficits [118]	Unknown	Unknown
PD	*Increase* Patients: substantia nigra [105]	*Upregulation* Patients: substantia nigra [50] *Treatment* Model mice: DuP697−Inhibition of dopaminergic neurotoxicity [121] Valdecoxib−Improvement of motor deficits [122] Patients: NSAIDs other than ibuprofen−No effect [123] Ibuprofen−Lower risk of development than non-use [124]	*Upregulation* Patients: mPGES-1 in substantia nigra [120] *Treatment* Model mice: Knockout−Suppression of the 6-OHDA-induced neurodegeneration [50]	Unknown	Unknown	*Treatment* Model cells: Butaprost (agonist) − Protection against oxidative stress [79]; Knockout − Enhanced microglia-mediated α-synuclein clearance [125]	Unknown	Unknown
MS	*Increase* Model mice: cerebral cortex, cerebellum, and spinal cord [127]	*Upregulation* Model mice: COX-1 and COX-2 in cerebral cortex, cerebellum, and spinal cord [92] *Treatment* Model mice: Indomethacin − Delay in the progression [126]	*Upregulation* Model mice: mPGES-1 in macrophages in spinal cord [128] Patients: mPGES-1 in macrophages in the brain [128] *Treatment* Model mice: Knockout − Improvement of clinical score [129]	Unknown	Unknown	*Function* Model mice: Enhanced generation of T helper 1 and helper 17 cells [130]	Unknown	*Function* Model mice: Enhanced generation of T helper 1 and helper 17 cells, and prevention of BBB invasion by these cells [130]

Aβ, amyloid β; AD, Alzheimer’s disease; ALS, amyotrophic lateral sclerosis; COX, cyclooxygenase; CSF, cerebrospinal fluid; EP, E-prostanoid receptor; HD, Huntington’s disease; mPGES-1, microsomal prostaglandin E synthase-1; MS, multiple sclerosis; 6-OHDA, 6-hydroxydopamine; PD, Parkinson's disease; 15-PGDH, 15-hydroxyprostaglandin dehydrogenase; PGE_2_, prostaglandin E_2_; ROS, reactive oxygen species

## Conclusions

Here, we summarize evidence on the pathophysiological role of PGE_2_ in neurodegenerative diseases, mainly focusing on ALS. PGE_2_ and its enzymes and receptors possess dual (destructive and neuroprotective) effects in multiple neurodegenerative disorders. Therefore, it should be noted that not only the local concentrations of PGE_2_ present in the lesion site but also the target cells and subtypes of activated receptors of PGE_2_ are involved in the disease, and further studies are required to clarify the pathophysiological role of PGE_2_ in neurons. A major challenge remains in adequately and sustainably delivering PGE_2_-specific inhibitors locally to target cells. This article provides new evidence on the spatiotemporal role of PGE_2_ synthetic and metabolic enzymes and receptors in the mechanism of cell death in ALS. We thus provide a new insight: selective inhibition of mPGES-1 and EP2 is potentially a new therapeutic strategy for patients with ALS.

## Data Availability

Not applicable.

## Abbreviations

15-PGDH: 15-Hydroxyprostaglandin dehydrogenase
6-OHDA: 6-Hydroxydopamine
AD: Alzheimer’s disease
ALS: Amyotrophic lateral sclerosis
Aβ: Amyloid-β
cAMP: Cyclic adenosine monophosphate
COX: Cyclooxygenase
cPGES: Cytosolic PGES
cPLA_2_: Cytosolic PLA_2_
CSF: Cerebrospinal fluids
EAE: Experimental autoimmune encephalomyelitis
EP: E-prostanoid receptor
fALS: Familial ALS
GFAP: Glial fibrillary acidic protein
HD: Huntington’s disease
IFN: Interferon
IL: Interleukin
LPS: Lipopolysaccharide
mPGES: Microsomal PGES
MS: Multiple sclerosis
NAC: *N*-acetyl-*L*-cysteine
NSAID: Non-steroidal anti-inflammatory drug
PD: Parkinson's disease
PGE_2_: Prostaglandin E_2_
PGES: Prostaglandin E synthase
PGH_2_: Prostaglandin H_2_
PGI_2_: Prostacyclin
PLA_2_: Phospholipase A_2_
ROS: Reactive oxygen species
SOD1: Superoxide dismutase 1
TDP-43: TAR DNA-binding protein-43
TNF: Tumor necrosis factor
